# Letters from Paris

**Published:** 1849-04-01

**Authors:** 


### CORRESPONDENCE FROM PARIS.

(*Communicated by* Dr. Sigmond.)

Medical science is still deserted, and its professors have in so many instances become statesmen, that we shall be almost led to imagine that the dissecting-room and the schools of anatomy in Paris are the cradles of politicians. One of them is now on his trial at Bourges, the indefatigable Raspail, whose freaks as a medical man were almost as extraordinary as those which have brought him before the present tribunal. It is deeply to be deplored that so much talent has been rendered abortive by the violence of political feeling.—The *Annales Medico-Psychologiques* are suspended, and there seems to be no prospect of their immediate appearance; this is very much to be regretted, for so many events have occurred, and so many cases worthy recording and examining, that science is losing some most important additions to the facts collected. Amongst them is the trial relating to the alleged insanity of M. Mortier, the ambassador at Turin, whose delusions were said to be such, that his wife and children were exposed to the constant dread of being destroyed. You will remember that he was confined first at Ivry, and then at a private establishment under the sanction of the Chancellor of the House of Peers, and a commission of inquiry; reports of various kinds were circulated, amongst others, that his insanity was to be attributed to his having taken large doses of belladonna for the alleviation of an agonizing tic douloureux, to which he was subject. After a long investigation, and a singular discrepancy of opinion exhibited by the medical men, the Court of Appeal has pronounced its opinion that the ambassador never was deranged; that all the suspicions of his wife's fidelity were mere misgivings of his mind, and that there was no case for the interference of the public authorities and the physicians. This singular judgment requires to be faithfully analyzed and explained, for much of mystery hangs over the whole affair.—M. Delasiauve, one of the physicians to the Bicêtre, is investigating with great attention the resources which we possess for the cure of epilepsy; his observations on the employment of sedatives are of considerable value. To valerian he gives the first place, though he does not speak of it quite in as sanguine language as did Tissot; a decoction of valerian given in doses of two wine glasses full, morning and evening, have produced a radical cure, but the medicine requires to be persevered in for a considerable length of time, otherwise it is of little avail; assafœtida decidedly moderates the violence of the access, but it does not seem to produce the same permanent good effect. The hydrocyanate of iron is found in some instances very beneficial, belladonna and digitalis are each serviceable, but Pluvrey of Lille prefers a combination of the two. The root of artemisia is occasionally useful; of liquid ammonia, according to the formula prepared by Martinet, he has had some good reason to speak, and will shortly give the results of his experience; camphor is found in those cases where the reproductive system is in a high state of excitement, as not unusually occurs in epileptics, to be of remarkable service; zinc, musk, castor, ambergris, have but little curative power; preparations of copper are to be but little confided in; nitrate of silver has lost the high character it once obtained; sulphate of quinine has also fallen into disrepute. He enters very minutely into the subject of the diet and exercise of the epileptic, preferring vegetable to animal food. It is to be regretted that the series of papers which this observant practitioner had prepared for the press are for the present suspended, for want of that encouragement which would have been at another period given to inquiries of such deep moment.—Baillarger is still proceeding with " his anatomical, physiological, and pathological researches upon the nervous system," but he has not yet completed the work, nor, in the present state of psychological science here, is there much prospect of it; a translation into English would, I have no doubt, be eagerly read by the English profession at large. His observations on the development of the brain form a remarkable addition to the knowledge we have obtained through Reil, Tiedemann, and Desmoulins; he arrives at the conclusion that this organ is developed from within, and that it increases by introsusception. His observations upon the hereditary nature of madness are invaluable; they are the result of a long series of indefatigable labours, and show how much he is in earnest in his inquiries after facts. He chose the three following questions for solution: Is the madness of the mother more frequently transmitted to the child than that of the father? In the case of hereditary madness, is the disease of the mother transmitted to a greater number of children than a similar malady in the father? Is madness more often transmitted from the mother to the girls, and from the father to the sons? No less than 600 cases have been investigated by him for the purpose of arriving at some distinct conclusions. It would be difficult for any one but an individual so advantageously placed as is Dr. Baillarger, at the Bicêtre, to have been able to collect such a quantity of statistic details as to have furnished him with proper data, for there are no documents existing which could be of much service; the only report of the kind being one which was made by Aubonel and Thore at the Bicêtre, and this of so limited a nature as to be of comparatively little value. From Dr. Baillarger's cases the result was, that out of 453 insane individuals, 271 had become so through the mother, if such an expression may be allowed, and 182 through the father, answering the first question by a large proportion passing through the female side; the second question was resolved from 271 families, in which the mother had transmitted the disease—in one infant in 203 cases; in two infants, 62; in three, five cases; in four infants, one; that is to say, that in one-fourth of the instances the mother's disease was shown in more than one case; out of 182 families, in which the disease descended from the father, a single child was diseased in 152 cases; two children, 36 times; three infants, four times; that is to say, that the madness appeared only in one-sixth of the instances, proving that the mother's malady affected the greater number of the descendants; the third question, whether the mother more frequently transmitted the disease to the girls, and the father to the sons, out of 346 children, he found that from the mother's side, 197 girls, and 119 boys were affected; and from 215, where the father had been the original source, 128 boys and 87 girls were affected, showing that the madness of the mother more frequently exhibited itself in the females than in the males; whilst, on the contrary, the madness of the father showed itself in the proportion of a third in the boys over the girls. Many are the physiological deductions which Dr. Baillarger has drawn from these inquiries, which, when the whole of his invaluable work is finished, will throw a great light upon the subject of hereditary madness.

M. Brierre de Boismont has read a paper on the employment of long-continued baths and sudden baths in the cure of acute paroxysms of madness, which possess some points of novelty, although the subject of cold affusion has been so long studied by those who have had the cure of the insane. The discussion upon the production of insanity in penitentiaries, where the silent system has been effectually carried out, has thrown very little light upon the matter, for there seems to be two parties in the Academy, one the adversaries, the other the partizans, of the system, who will allow each to have but little real knowledge of the matter; indeed, there seems as yet but scanty information on which to arrive at a conclusion. The ordonnance at Vannes was put into force on the 10th of May, 1839; there were 300 prisoners upon an average annually, of whom there have died each year twenty-nine, and eight have become deranged. Among the exciting causes of these numerous casualties must be reckoned the absolute silence, enjoined in all the common occupations of life, the chagrin which is thus necessarily produced, and the want of exercise. There is to be a general discussion on the subject, and a Report made to the Government. There have been several cases of madness, produced by baffled ambition, during the late struggles for power, amongst a class of persons who could scarcely ever have contemplated rising above the ordinary ranks of society, but who suddenly saw themselves on the high road to preferment. Again, many individuals have been similarly affected, who, for a short time, were placed in positions of trust, and have again, by the changes that have so frequently occurred, become deprived of their short-lived prosperity. Such cases must, however, occur in all such disjointed states of society as those we have lately witnessed, and even amongst a people more excitable than the French. It is said amongst the medical men, that in Italy, especially in Rome, there have been a vast number of instances of sudden political frenzy, and that the establishments for the reception of the insane, both public and private, have been, within a very short time, thronged with persons before unsuspected of being at all predisposed to the malady. This is especially the case at Venice, where the establishment of the greatest importance is under the immediate government of the monks, who are, for the most part, jealous of all medical interference.

It is singular that stramonium, both the datura and the fastuosa, should have an influence upon the state of excitement which follows upon the exaltation of political ideas, yet there are numerous instances on record which leave very little doubt of the fact. One young lad, who was under the care of Dr. Moreau, at the Bicêtre, furnishes an admirable example. A letter of his to his physician, written in the true style of the day, will show what the nature of his case was:— "Give me my liberty, that I may actively work for the overthrow of all kings; I wish to regenerate the human species; I am destined to die at the head of a powerful republic. From the age of eighteen, I have had the idea, like Romulus, of building a city in the forests of Lorraine, which is my native country." He was occupied principally in writing letters to the Pope, to Louis Philippe, and to Prince Metternich—in the clouds he saw myriads of armed men, whilst the voice of angels foretold his future grandeur. He was constantly trying to tie handkerchiefs to the tops of the trees, to correspond with his partisans. He was submitted to the action, for ten days, of datura, which produced, in the dose of two grains, somnolence, constriction of the throat, pressure at the temples, then melancholy—he saw spectres chanting a funeral dirge around his bed at night; but he began progressively to mend, and to perceive his errors; he began to take an interest in playing cards; but on one day, he took an enormous dose of the pills, went through the symptoms of intoxication, but recovered the use of his senses from that period, laughed at his errors, and has become satisfied with things as they are. Many similar instances have been recorded, although the connexion between the state produced by the drug, and the remedy itself, is altogether incapable of being explained. At a meeting on the 2nd of May, last year, of the National Academy, M. Belhomme undertook the investigation of the influence of political events upon the development of mental alienation, and his paper was referred to a committee, composed of M. Ferrus, Talvet, and Gueneau de Massy; doubtless they have had a still wider field for their labours than when they commenced, and will be enabled to furnish us with matter well worthy the deepest investigation. It was in Belhomme's opinion, that individuals predisposed were those thus affected—that the form was acute, therefore more easy of cure; that the sedative treatment was indicated, particularly prolonged bathing, with cold affusion upon the top of the head, derivatives to the intestinal canal and to the surface—and that cautious moral treatment was of the utmost importance.—M. Moreau Christophe, Inspector-general of Prisons, is very busily occupied in the penitentiary system, and the opinion that was brought before the Academy of Medicine, that in a given number of prisoners, there is a greater number of madmen than amongst an equal number of honest persons, is undergoing an examination. M. Lelut has shown, from statistic facts, that the number of insane is four, five, and even six times greater in prisons than in the unconfined population. During the period of incubation of insanity, a vast number of the crimes which are committed, occur, and the disease is only fully evinced when the judgment is pronounced. This wakes up in the mind new sensations, which are for the first time associated with the horrors of punishment, and then the intellect becomes completely disordered. The battle of conscience, the struggles of remorse, especially amongst females, together with the privations which are endured, all become a frequent cause, where a predisposition exists, of the development of the malady. It not unfrequently occurs, that prisoners full of strength and courage, both before and during the trial, fall into a sort of moral prostration after condemnation, and even after acquittal, and then are perceived symptoms which would render the punishment of their delinquencies the worst species of punishment. How far simulated madness may sometimes deceive those who are earnest in their inquiries, and eager for the exercise of humanity, yet remains to be judged of. It certainly reflects very highly upon the character of the benevolent persons who are interesting themselves in these matters, that they have not permitted the uncertain and unhappy state of the country from interfering in their pursuits.—Several cases of infanticide have lately occurred in Paris, and some very grave reflections have arisen out of them, and attempts to demonstrate, that in every such instance, there must be madness present. A poor girl, having been condemned for fifteen years to hard labour, for the destruction of her child, the seducer being her own master, has called forth some very long arguments. It is asserted, that as the Foundling Hospital is open to every one here,—and that this fact is well known is proved by the average admission of three infants daily,—that nothing but madness could lead a parent to the murder of its offspring. People of the better condition constantly send their newly-born babes there—an example which was not only set, but boldly avowed, by Jean Jacques Rousseau, and therefore there is not that frightful condition which might plead an excuse. This subject would be one of some difficulty, even to allude to, in the state of English morality, but here there is less of delicacy and of feeling; the consequence is, that the subject is likely to be warmly discussed.—The Societé-Medico-Psychologique, which has been attempted to be founded, is again constituted; in its prospectus, it speaks with considerable pleasure of the *British "Journal of Psychological Medicine and Mental Pathology,"* congratulating the European commonwealth of science upon its appearance, and thanking Dr. Forbes Winslow for the example he has set, and expressing the hope that it will be followed all over the civilized world. It looks, however, to the French publication as the parent of the journal, and only regrets that times and circumstances should impede the progress of the *Annales Medico-Psychologique.* Although the list of the members has been promised, it has not yet appeared, nor the regulations; but as they have to be submitted to the Minister of Public Instruction, it may be some time before they are made known, more especially if there are to be changes of administration. It is a matter of regret that the Minister of Public Instruction is not a permanent officer, for just as he becomes conversant with science and its followers, he has to make way for a substitute; and in France, a change of ministers requires weeks to accomplish, so as to place matters upon their former foundation. An example of such a society in Paris ought to awaken attention in England, at any rate a library should be founded, devoted to works upon the study of the mind.

